# Public policies on healthcare-associated infections: a Brazil and UK case study

**DOI:** 10.11606/S1518-8787.2017051000315

**Published:** 2017-12-04

**Authors:** Maria Clara Padoveze, Sara Melo, Simon Bishop, Vanessa de Brito Poveda, Carlos Magno Castelo Branco Fortaleza

**Affiliations:** I Universidade de São Paulo. Escola de Enfermagem. Departamento de Enfermagem em Saúde Coletiva. São Paulo, SP, Brasil; II Queen's University Belfast. Queen's Management School. Belfast, Northern Ireland, United Kingdom; III University of Nottingham. Nottingham University Business School. Nottingham, United Kingdom; IV Universidade de São Paulo. Escola de Enfermagem. Departamento de Enfermagem Médico Cirúrgica. São Paulo, SP, Brasil; V Universidade Estadual Paulista. Faculdade de Medicina de Botucatu. Departamento de Doenças Tropicais. Botucatu, SP, Brasil

**Keywords:** Cross Infection, prevention & control, Infection Control, organization & administration Public Health Policy, Public Health, history

## Abstract

To summarize the historical events and drivers underlying public policy for the prevention and control of healthcare-associated infections in Brazil and in the United Kingdom. In doing so, the article aims to identify lessons and recommendations for future development of public policy. The analysis is based on a historical overview of national healthcare-associated infections programs taken from previously published sources. Findings highlight how the development of healthcare-associated infections prevention and control policies followed similar trajectories in Brazil and the United Kingdom. This can be conceptualized around four sequential phases: Formation, Consolidation, Standardization, and Monitoring and Evaluation. However, while we identified similar phases of development in Brazil and the United Kingdom, it can be seen that the former entered each stage around 20 years after the latter.

## INTRODUCTION

Healthcare-associated infections (HAI) are a public health concern worldwide. According to the World Health Organization (WHO), nations should have HAI Programs (HAIP) at national and local (healthcare settings) levels^32^. A national program is intended to regulate, provide guidance, promote, and supervise compliance with regulations^32^. The HAIP at local level aim to prevent the occurrence of HAI in patients, healthcare workers, and visitors. Well established HAIP are required to deal with outbreaks.

The HAIP vary among countries, resulting in differences in outcomes of HAI rates. Therefore, there is scope for considerable cross-national learning. Studying the processes by which HAIP have been developed in different countries over time will help us to understand the trajectory of change and to identify areas for improvement.

The objective of this study was to identify the main drivers and events underlying the development of public policies on HAI prevention and control (P&C) in two countries and draw out policy lessons from historical experience. Brazil and the United Kingdom (UK) were selected as both have healthcare systems informed by the Beveridge model, involving government funding of healthcare services, financed by general taxation^15^ ^,^ ^27^.

We conducted a historical overview of national HAIP, by an open-ended review of published sources describing key policy developments. From this literature, we identified four sequential phases for the development of HAI P&C policies at national level. Formation (development of infection prevention techniques and practices); Consolidation (acknowledgement of HAI as a public health problem and development of the initial proposals of national HAIP); Standardization (consolidation of HAIP and establishment of nation-wide regulations); Monitoring and Evaluation (full establishment of HAIP at national level including mechanisms for measurement; recognition of HAI as a relevant patient safety issue, focus on continuous quality improvement and cost savings). As presented below (summarized on Figure), elements of each of these four phases were seen in both countries, albeit in different periods.

**Figure:**
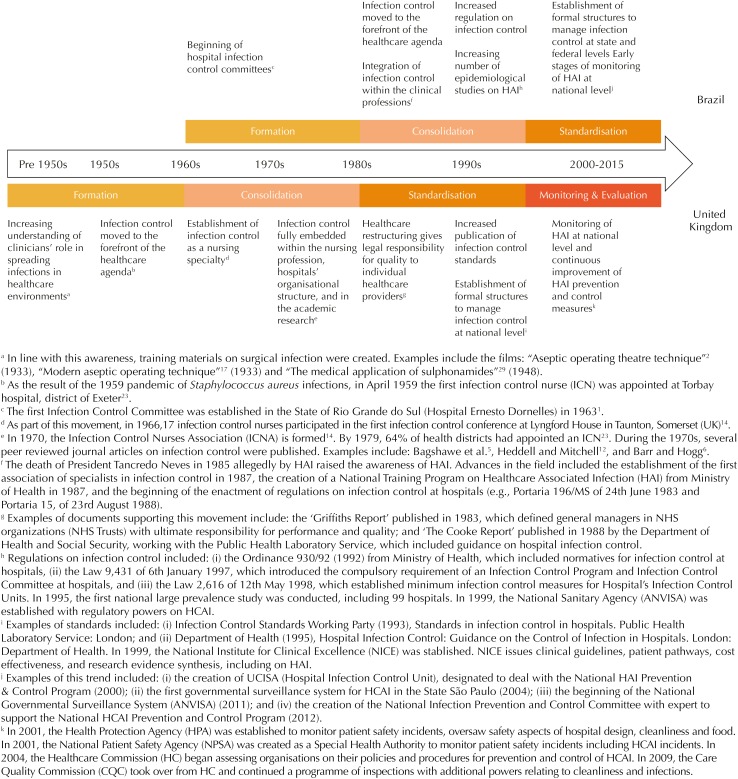
Evolution of healthcare-associated infection (HAI) prevention and control in the United Kingdom and Brazil until 2015.

### United Kingdom

#### Formation.

In the early of the 20th century, the UK saw significant scientific discoveries on P&C, despite considerable regional variations in healthcare practices. The inauguration of the National Health Service (NHS) in 1948^31^ saw the beginning of national initiatives to reduce HAI, and epidemics of *Staphylococcus aureus* during the 1950s brought these issues to wider public attention.

#### Consolidation.

During the 1960s, the P&C of HAI was placed as an essential component for healthcare practices. This included the formal establishment of infection control as a nursing specialty, as well as the inauguration of working groups, conferences, and journals. These activities helped to raise the concern about HAI at the national and politics level.

#### Standardization.

Changes to national policy related to healthcare organizations settled legal responsibility of individual providers for effective HAIP. In addition, publications of detailed national guidelines, and reporting mechanisms as scientific and professional communities increasingly gained a place in policy level decision making, such as through the National Institute of Clinical Excellence. Together, these developments raised HAIP compliance.

#### Monitoring and Evaluation.

Currently, HAIP are well embedded into national healthcare activities and organizations; guidance on P&C and surveillance systems are under the Public Health England authority. Each country within the UK has its own HAIP led by national public health bodies based on similar principles and practices. Practice is shaped by national evidence-based guidelines^16^ and broad principles of best practice with the aim of integration into routine practice. A national surveillance program for HAI covers *Staphylococcus aureus, Escherichia coli, Clostridium difficile*, surgical site infections, and patients with urinary catheter, producing annual reports. In 2015, there was an increased policy focus on antibiotic resistance and antibiotics over prescription following reports of continued overuse^24^. This also includes providing operational guidance for health teams regarding the prevention and management of HAI for all healthcare settings including the National Health Service and the private sector. As a result, a downward trend in HAI rates^13^ is reported, from point prevalence surveys.

### Brazil

#### Formation.

The start of HAIP in Brazil dated of the 1960s, with the formal creation of the first Infection Control Committee (ICC) in the state of Rio Grande do Sul^1^. By the end of the political military regime, the fact that the first civil president in 1985 supposedly died due to HAI fostered a visibility of the role of ICC. At the time, HAI P&C initiatives were led by individual professional associations. In 1987, the São Paulo Association of Infection Control and Epidemiology (APECIH - *Associação Paulista de Epidemiologia e Controle de Infecção Relacionada à Assistência à Saúde)* was founded, offering a central point of focus for the P&C of HAI, disseminating scientific knowledge and bringing together professional activities in the area. In the same year, the National Association of Professionals (ABIH - *Associação Brasileira dos Profissionais em Controle de Infecção e Epidemiologia Hospitalar)* was created and subsequently, the Ministry of Health began a national training program^a^.

#### Consolidation.

In the decade of 1980s, the creation of the Unified Health System (SUS) established health as a right in the country^9^. Subsequently, the 1990s saw increasing consolidation of HAI P&C at the policy level. National regulations were reviewed, culminating in a law making it compulsory for all hospitals to establish an ICC. The first national HAI prevalence study was carried out in the 1990s, although a follow-up study was not conducted for nearly 20 years^21^ ^,^ ^26^.

#### Standardization.

Standardization in Brazil can be seen to gather pace following outbreaks of HAI in the late 1990s. This includes the 1996 outbreak of intoxication by cyanobacteria at a hemodialysis unit in Caruaru, Northeastern Brazil, due to the use of improperly treated water, leading to 126 HAI cases and 60 deaths^25^. Further outbreaks of fast growing *Mycobacteria* ^b^ and multidrug resistant microorganisms have triggered the development of further regulations by the National Sanitary Agency (ANVISA)^4^ ^,^ ^7^ ^,^ ^18^ ^,^ 20 ^,^ ^30^. Standardization was strengthened by the 1998 Ministry of Health regulation defining the role of national, state, and municipal surveillance systems to monitor and evaluate P&C of HAI nationwide (Figure).

Currently, the coordination of the national HAIP is carried out by ANVISA, which sets the basic elements for P&C of HAI, including written guidelines and a surveillance system. This system is targeted at catheter-related bloodstream infections at the intensive care units and high risk nurseries and surgical site infection^c^. A system for outbreak notification^d^ is in place and the National HAIP is publicly available^e^.

Since 2000, ANVISA established working groups involving experts to develop guidelines on P&C of HAI and also created the National Infection P&C Committee to support the national HAIP. Recently, Brazil initiated a patient safety program at the national level^19^.

Data from the national surveillance system shows a huge variation in HAI rates among the states^f^. There is also heterogeneity at the local level, with some hospitals having well established HAIP, while others have almost no initiatives in this area^19^ ^,^ ^21^.

## FINAL CONSIDERATIONS

Across the two countries, we found similarities in the phases of HAI public policy, though, when compared to the UK, the start of each phase in Brazil took place around 20 years later. Of note, our analysis sought to identify trends in the HAIP at national level, rather than considering local practices, which are likely to vary greatly.

This article focuses on the development of HAI public policies in Brazil and in the UK. However, other factors may have influenced the attention surrounding HAI. From the start, in the past decades, there were significant differences in the economic, cultural, and political landscape of the two countries. Second, although both healthcare systems are informed by the Beveridge model, the way each system is organized may affect the perspectives of HAI prevention^10^. The NHS began in the UK in 1948, while in Brazil, the SUS was not fully developed until the 1990s. Whereas in the UK free and comprehensive healthcare provision dates from the late 1940s, in Brazil, health as a citizen's right and a duty of the state was only recognized with the promulgation of a new constitution in 1988. The principles of universality, comprehensiveness, and social participation adopted by the SUS did not exist before^22^. This perception of citizen's rights is probably associated with the raising of awareness of the importance of P&C of HAI. Hence, this can be a relevant driven force to promote P&C policies. Worldwide, healthcare systems are evolving and encompassing more complex and diverse environments of care (e.g. primary and secondary care, community care, etc.)^10^ ^,^ ^15^. Therefore, strategies to prevent HAI should also evolve in order to be able to deal with this diversity.

Findings also suggest that additional international trends shaped HAI public policy developments. Namely, the cultural and political affinities between the UK and the USA facilitated the sharing of evidence and practice between the two countries. For example, the US SENIC study (1980)^11^ demonstrated that effective programs could reduce at least 30% of HAI and gained widespread policy attention in the UK. However, we saw the influence from international studies being exerted much later in Brazil.

Evidence suggests that Brazil only developed scientific critical mass on the issue of HAI in the 1990s. Since then, the exchange between Brazilian and international researchers in this field promoted knowledge transfer and discussions on the feasibility of translating to the Brazilian context techniques and systems applied elsewhere^28^. Alongside the development of HAI research in Brazil, scientists have been asked by ANVISA to contribute in the Standardization process. In Brazil, we now see a better synchronicity between the scientific understanding and the HAI public policy that has allowed the country to arrive at the stages of Consolidation and Standardization. Further, stemming from our historical comparative overview, we would suggest that Brazil is likely to enter into a phase of Monitoring and Evaluation, which is already established in the UK. The influence of international bodies such as the WHO may have played a role in advancing the HAI subject, particularly in developing countries.

Another important driver of the development of HAIP in both countries was the attention brought by the occurrence of relevant outbreaks. In the two countries outbreaks and subsequent media campaigns led to periods of increased public and political sensitivity to the issue. The raising of awareness of legal and civil rights for the provision of quality and safe healthcare appeared to be a key pressure point for the processes of Standardization.

Patient safety has been accepted as a worldwide problem to be tackled and HAI rates are recognized as a key indicator of quality of care. Safer care can be seen as a shared goal in high and middle-income countries, and our study recognizes points of common development in HAIP in the UK and Brazil. Nevertheless, there remain significant differences between the two countries. In comparative terms, Brazil has to overcome multiple political and economic challenges in order to advance universal healthcare provision^8^. Indeed, for many middle-income countries, basic access to healthcare continues to be a problem for some portions of the population, and wherever this is the case, the prevention of HAI may be seen as a secondary concern^3^. A good balance between healthcare access, economic sustainability, and patient safety is the challenge for healthcare systems worldwide.

The analysis of the UK experience highlights several potentially useful lessons for health systems that have not yet reached the Monitoring and Evaluation phase. Amongst them, the need to consider HAI as a public health problem, consistent dissemination of evidence based guidelines, integration of scientific updates in the clinical practice, setting up of guidance addressed to different types of healthcare settings, and monitoring of epidemiologically relevant pathogens. Finally, citizens engagement in the HAI policy making is highly desirable.

## Footnotes

a National Training Program on HCAI - The Brazilian Ministry of Health (MoH) used the strategy of “training the trainers”. Initially, a centralized training program occurred in Brasilia, Federal District. After that, the training program was carried out at national level using voluntary training centers and their staff. All didactic material was provided by the MoH.

b Agência Nacional de Vigilância Sanitária. Relatório descritivo de investigação de casos de infecções por micobactérias não tuberculosas de crescimento rápido (MCR) no Brasil no período de 1998 a 2009. Brasília (DF): ANVISA; 2011[cited 2011 Nov 25]. Available from: http://www.anvisa.gov.br/hotsite/hotsite_micobacteria/relatorio_descrito_mcr_16_02_11.pdf

c Agência Nacional de Vigilância Sanitária. Segurança do paciente. Brasília (DF): ANVISA; s.d. [cited 2015 May 30]. Available from: https://www20.anvisa.gov.br/segurancadopaciente/

d Ministério da Saúde (BR), DATASUS. Formsus: versão 3.0: Notificação de agregado de casos e surto em serviços de saúde. Brasília (DF): DATASUS; s.d. [cited 2015 May 30]. Available from: http://formsus.datasus.gov.br/site/formulario.php?id_aplicacao=8934

e Agência Nacional de Vigilância Sanitária. Segurança do paciente: PNPCIRAS 2016-2020 Brasília (DF): ANVISA; s.d. [cited 2015 May 30]. Availale from: https://www20.anvisa.gov.br/segurancadopaciente/index.php/publicacoes/item/pnpciras-2016-2020

f Agência Nacional de Vigilância Sanitária. Segurança do paciente. Brasília (DF): ANVISA; s.d. [cited 2015 May 30]. Available from: https://www20.anvisa.gov.br/segurancadopaciente/
